# Detailed NMR, Including 1,1-ADEQUATE, and Anticancer Studies of Compounds from the Echinoderm *Colobometra perspinosa*

**DOI:** 10.3390/md7040565

**Published:** 2009-11-12

**Authors:** Anthony D. Wright, Jonathan L. Nielson, Dianne M. Tapiolas, Cherie A. Motti, Simon P. B. Ovenden, Philip S. Kearns, Catherine H. Liptrot

**Affiliations:** 1 Australian Institute of Marine Science, PMB No. 3, Townsville MC, Townsville, 4810, Australia; E-Mails: d.tapiolas@aims.gov.au (D.M.T.); c.motti@aims.gov.au (C.A.M.); p.kearns@aims.gov.au (P.S.K.); 2 Present address: University of Hawaii, College of Pharmacy, 34 Rainbow Drive, Hilo, HI 96720, USA; 3 Present address: ACD Labs UK, Building A, Trinity Court, Wokingham Road, Bracknell, Berkshire, RG42 1PL, UK; 4 Present address: Defense Science and Technology Organisation, 506 Lorimer Street, Fishermans Bend, Port Melbourne, Victoria 3207, Australia

**Keywords:** 1,1-ADEQUATE, NMR, marine natural products, echinoderm, Colobometra perspinosa, anticancer

## Abstract

From the dichloromethane/methanol extract of the crinoid *Colobometra perspinosa*, collected south east of Richards Island (Bedara), Family Islands, Central Great Barrier Reef, Australia, 3-(1′-hydroxypropyl)-1,6,8-trihydroxy-9,10-anthraquinone [one of the two stereoisomers of rhodoptilometrin, (**1**)], 3-propyl-1,6,8-trihydroxy-9,10-anthraquinone (**3**), 2-[(phenylacetyl)amino]ethanesulfonic acid (**4**), and 4-hydroxybutanoic acid (**5**) were isolated. Comparison of ^1^H- and ^13^C-NMR data for rhodoptilometrin (**1**) with those reported in the literature showed significant differences for some resonances associated with rings A and C. In an attempt to provide accurately assigned ^1^H- and ^13^C-NMR data, as well as to confirm the structure of **1,** a thorough NMR investigation of this compound was undertaken. Measurements included: concentration dependent ^13^C, 1D selective NOE, HSQC, HMBC and 1,1-ADEQUATE. The NMR data for **4** and **5** are reported here for the first time, as is their occurrence from the marine environment. The *in vitro* anticancer activity of the original extract was found to be associated with **1**, **3** and **5**.

## 1. Introduction

Numerous marine organisms are known to produce secondary metabolites as defense mechanisms [1–4] which ultimately prove to have other interesting biological activities [5–7]. Many of these compounds are brightly coloured. Crinoids are known producers of brightly coloured, typically orange red pigments that are predominantly anthraquinoid in nature. The NMR data for these compounds are difficult to assign and as a result are often incorrectly assigned [8–10]. NMR techniques that are not routinely used need to be applied to resolve assignment issues for this class of compounds [11]. During a screening campaign to identify marine extracts with whole cell anticancer activity, a dichloromethane/methanol (DCM/MeOH) extract of a specimen of the crinoid *Colobometra perspinosa*, collected from south east of Richards Island (Bedara), Family Islands, Central Great Barrier Reef, Australia, was found to be active. Subsequent bioassay and ^1^H-NMR guided fractionation of the MeOH soluble fraction led to the isolation of compounds **1**, **3**, **4** and **5**. Outlined in this publication are details of the structure elucidation of **1**, **3**, **4** and **5**, as well as a detailed discussion of the ^13^C-NMR data associated with **1** and **3**, together with their cytotoxicity, as assessed in a series of anticancer cell based assays.

## 2. Results and Discussion

**Figure 1.**
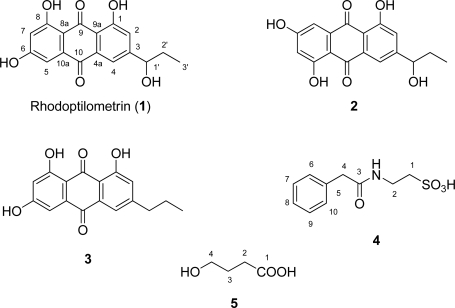
Structures of compounds 1–5.

**Figure 2.**
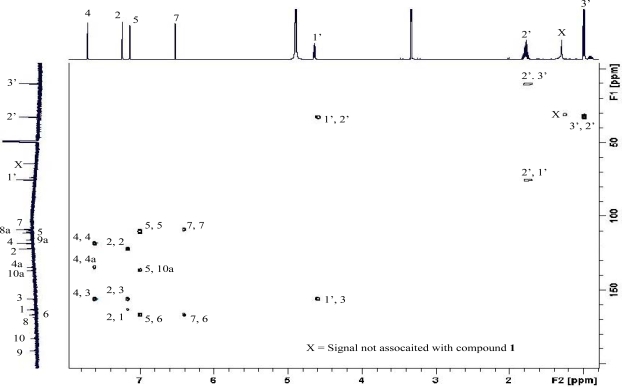
1,1-ADEQUATE spectrum of **1** (600 MHz basic frequency, CD_3_OD).

Rhodoptilometrin (**1**) was isolated as an orange-yellow microcrystalline solid with a molecular weight corresponding to the molecular formula C_17_H_14_O_6_; the molecule had eleven double bond equivalents of unsaturation. Analysis of the NMR data for **1** (Table 1) showed it to have 6 × C=C, and 2 × C=O as the only multiple bonds within the molecule, so **1** was tricyclic. Other structural features of the molecule, gleaned from its spectroscopic data, were the presence of two pairs of meta-coupled protons and a 1-hydroxypropyl moiety. These deductions indicated **1** to be a tetra-substituted anthraquinone, where the substituents were three hydroxyl groups and a 1-hydroxypropyl moiety. From ^13^C- and ^1^H-^13^C HMBC data (see Table 1) it was possible to establish the substitution pattern of the anthraquinone as shown in Figure 1. Comparison of the NMR data in Table 1 for **1** with those reported for rhodoptilometrin (see Table 1 and Table 2) [8–10], raised doubts about the structure, in particular the ^13^C-NMR resonances for C-4, 5, 6, 8a and 9a, associated with rings A and C of **1**, and the ^1^H-NMR data for H_3_-3′. If the reported structure of **1** was incorrect, the only other structure that fits all of the available data is the regioisomer **2**. In an attempt to resolve this regio-chemical issue and provide reliable NMR data, a 1,1-ADEQUATE spectrum [11] (see Figure 2 and Table 1), as well as ^13^C-NMR spectra at various concentrations (see Table 2), were recorded, and all of the NMR data re-examined. There was a slight influence on ^13^C-NMR chemical shifts with changing concentration (see Table 2), however, nothing to the extent that would account for the differences seen between resonances associated with C-4, 5, 6, 8a and 9a reported in the literature [10] and current values. The 1,1-ADEQUATE cross-peaks observed for **1** between H-4 and C-3, 4, and 4a, between H-2 and C-1 and 3, and between H-1′ and C-2′ and 3, (see Figure 2 and Table 1), coupled with the HMBC cross-peaks observed between H-4 and C-1, 2, 9 (weak), 9a and 10, defined ring C, the 1-hydroxypropyl moiety, and C-10, as shown in **1**. Also 1,1-ADEQUATE cross-peaks were observed between H-5 and C-6 and 10a, and between H-7 and C-6, together with HMBC cross-peaks observed between H-5 and C-6, 7, 8, 8a, 9 (weak) and 10, and between H-7 and C-5, 6, 8, 8a and 9 (weak). This defined ring A and C-9, thus completing ring B of the molecule and confirming the planar structure. Based on this analysis, the compound isolated in this study was assigned as **1**. The specific rotation of **1** measured at 589 nm was -22, which is in agreement with that reported for rhodoptilometrin by Lee *at al.* [10]. Careful consideration of the C-4, 5, 6, 8a and 9a ^13^C-NMR chemical shift differences observed between our **1** and that reported by Lee *et al.* [10], highlighted that, with the exception of C-6, these carbons would possibly be influenced by tautomeric effects. This raised the issue of pH and whether the compounds were in the same form prior to NMR analysis. In our case, all samples were acid and base free prior to measurement as were NMR solvents used for the measurement. Adding formic acid or sodium hydroxide to our NMR sample in CD_3_OD did not alter the chemical shifts of these carbons. Compound **1** was also isolated from a more recent collection of *Colobometra perspinosa* collected from Coombe Island in the Family Islands, Central Great Barrier Reef, Australia in 2005. It seems likely that the compound isolated and characterized by Lee *et al.*, [10] probably is rhodoptilometrin (**1**), but in the absence of more details concerning isolation and measurement conditions (e.g., temperature, concentration and pH) this cannot be stated with certainty.

Together with rhodoptilometrin (**1**), 3-propyl-1,6,8-trihydroxy-9,10-anthraquinone (**3**), or dehydroxyrhodoptilometrin, was also isolated and characterized spectroscopically. From comparison of the ^1^H- and ^13^C-NMR data for **1** with that obtained for **3**, together with the accurate mass data, it was evident that **3** was also a tetra-substituted anthraquinone with three hydroxyl groups and an aliphatic moiety at C-3. The differences in the ^1^H- and ^13^C-NMR chemical shifts of the aliphatic substituent between **1** and **3** clearly revealed the presence of a propyl moiety at C-3. For **3**, complete assignment of ^1^H- and ^13^C-NMR data are provided for the first time; see Experimental Section and Table 1.

The conjugate of phenylacetic acid and taurine, phenylacetyltaurine, or 2-[(phenylacetyl)amino]ethanesulfonic acid (**4**) was also isolated from the MeOH extract. The structural elucidation of this molecule was relatively straightforward once the elemental composition was determined from accurate mass measurement (C_10_H_12_NO_4_S). It was possible to deduce two molecular fragments from the ^1^H- and ^13^C-NMR data of **4** (see Experimental Section); the phenylacetic acid (amide) moiety, an ethyl chain with a terminal nitrogen at C-2, and a heteroatom, unlikely to be oxygen or nitrogen, at C-1. With the accurate MS measurement in hand it was evident that the group at C-1 was a sulfonic acid and that **4** was the conjugate from the reaction between phenylacetic acid and taurine. Surprisingly, this appears to be the first report of this entity as a natural product from the marine environment, even though it is encountered fairly often in metabolic studies involving the use of phenylacetic acid [12,13].

The final compound to be identified in this study was 4-hydroxybutanoic acid or gamma-hydroxybutyric acid (GHB, **5**). The structure elucidation of this molecule was routine. From the ^1^H- and ^13^C-NMR data of **5** (see Experimental Section), it was evident that the molecule was composed of a butyl chain terminating at one end with an OH (C-4, δ_C_ 68.5 ppm); and at the other with a carboxylic acid or amide (C-1, δ_C_ 177.8 ppm); these deductions were confirmed by routine low resolution MS of **5**, and showed it to be the free acid and not the amide. The discovery of **5** in a marine organism is in itself not surprising, as the molecule is a simple one and commonly found in low concentrations in mammals [14]. What does make the discovery interesting is the colourful history associated with **5**. It is a naturally occurring substance, probably synthesized from gamma-aminobutyric acid, found in the central nervous system (CNS) of mammals and thought to act as a neurotransmitter. It is also found in other organs such as the liver, kidneys, and heart as well as in bones. As a drug it is most commonly used in salt form [sodium gamma-hydroxybutyrate (Na-GHB, sodium oxybate) or potassium gamma-hydroxybutyrate (K-GHB)], and is used in the treatment of insomnia, depression [15], alcoholism [16], historically as an anesthetic, and for sleep disorders [17]. Its real notoriety comes from it being represented as a date rape drug, often being referred to as liquid ecstasy despite its unrelated effects [18].

Compounds **1**, **3**–**5** were screened for their *in vitro* anti-cancer activity against three human tumour cell lines [MCF-7 (breast-pleural effusion adenocarcinoma), SF-268 (CNS-glioblastoma), and H460 (lung-large cell carcinoma)], the results of which are shown in Table 3. From the bioassay data it can be seen that **1**, **3** and **5** all demonstrate some non-selective activity towards the three cell lines used for the testing, and that functionality in the side chain of **1**, compared to **3**, appears not to influence this activity. The activity observed for **3** is consistent with previous findings for its sulfate derivative [10]. Compound **4** appears to be weakly active in only one of the three cell lines (MCF-7).

## 3. Experimental Section

### 3.1. General experimental

C18 flash vacuum chromatography was performed using Phenomenex C18 (50 μm). HPLC was performed with a Shimadzu HPLC system consisting of a Shimadzu SCL-10Avp system controller equipped with a Shimadzu LC-10AT pump, Shimadzu SPD-M10Avp photodiode array detector, Shimadzu FRC-10A fraction collector and Shimadzu SIL-10A auto sampler. All HPLC data were collected using the Shimadzu Class-VP data collection package installed on a Dell Pentium 4 PC running Windows 2000. IR spectra were measured on a Nicolet Nexus FTIR. Optical rotations were recorded on a Jasco P-1020 polarimeter. All NMR spectra were measured on a Bruker Avance 600 MHz NMR spectrometer complete with cryoprobe. NMR spectra were referenced to residual ^1^H and ^13^C resonances in the deuterated solvents. Low resolution mass spectral data were measured on a Bruker Daltonics Esquire 3000 plus mass spectrometer, complete with Agilent 1100 HPLC system comprising of pump, PDA and autosampler. Accurate mass spectrometric data were measured on a Bruker BioApex 47 FT mass spectrometer. All other details as previously published [19].

### 3.2. Animal material

The original sample of the crinoid *Colobometra perspinosa* (family Colobometridae), was collected from south east of Richards Island (Bedara), Family Islands, Central Great Barrier Reef, Australia, at a depth of 12 m, on June the 12^th^, 1989 and immediately frozen and stored at −20 °C. A voucher specimen is lodged with Queensland Museum, accession number G212468. The sample was collected under GBRMPA permit G88/354 and Queensland Fisheries permit 1780. The second collection of the crinoid was made from Coombe Island, Family Islands, Central Great Barrier Reef, Australia on June the 27th, 2005 and immediately frozen and stored at −20 °C. A voucher specimen (27124) is lodged at AIMS.

### 3.3. Bioassay

The cell lines SF-268, MCF-7 and H460 were grown in RPMI 1640 medium with l-glutamine supplemented with 5% foetal bovine serum and maintained in a humidified incubator at 37 °C with 5% CO_2_. Cells were plated in 96 well microtitre plates at a seeding density of 5,000 cells per well in 100 μL medium and allowed to attach for 24 hours. Natural product samples were solubilised in DMSO and serial dilutions prepared in medium. These were added to the cells so that the final doses ranged from 250 μg/mL to 3 μg/mL. The plates were returned to the incubator. Total cellular protein was used as an indicator of cell number and was measured at 0 hours and 48 hours after sample addition using the sulforhodamine B (SRB) assay. Cells were fixed by addition of 30 μL of 50% TCA for 30 min at 4 °C, rinsed five times in running water then air dried before staining with 50 μL 0.4% SRB in 1% acetic acid for 30 mins at room temperature. Plates were washed in five changes of 1% acetic acid and air dried. SRB dye was solubilised in 10 mM Tris (100 μL) and plates read on a Wallac Victor Plate reader with excitation at 540 nm and emission at 590 nm. The concentration at which growth was inhibited by 50% (GI_50_) was determined by comparing the dose response curves of sample treated values to those of vehicle only control (100% growth) and Time 0 readings (zero growth). Taxol and staurosporine were used as positive controls and medium only and untreated wells were used as negative controls.

### 3.4. Extraction and isolation

The organic solubles (3.37 g of organic extract, obtained employing repeated extraction with 1:1 DCM-MeOH) obtained from the crinoid were filtered through a plug of reversed phase C18 silica using MeOH as eluent. The MeOH was removed under reduced pressure and the resultant filtrate subjected to preparative RP-HPLC (9.5 mL/min, gradient elution from 10% MeCN:H_2_O (+0.1% formic acid) to MeCN (+0.1% formic acid); column 250 × 20 mm RP Luna C18 (2), Phenomenex) over 80 mins, to yield 34 fractions. Of the 34 fractions two, fractions 16 and 17, were found to be active in the bioassay. ^1^H-NMR analysis of all fractions showed fractions 9–11 to be **4** (16.7 mg, 0.5% organic extract), fractions 16 and 17 to be **1** (75.3 mg, 2.2% organic extract), fraction 24 to be **3** (9.5 mg, 0.3% organic extract), and fraction 34 to be **5** (27.7 mg, 0.8% organic extract).

### 3.5. 3-(1’-Hydroxypropyl)-1,6,8-trihydroxy-9,10-anthraquinone (Rhodoptilometrin, **1**)

An orange-yellow microcrystalline solid. [α]^20^_D_ -22 (*c* 0.1, MeOH); IR (film) ν_max_ 3420, 1660, 1280 cm^−1^; UV (PDA, MeOH) λ_max_ 512 nm; ^1^H- (600 MHz, CD_3_OD), and ^13^C- (150 MHz, CD_3_OD) NMR data see Table 1; HRESIMS *m/z* found 313.0710 for [M-H]^−^ (calcd for C_17_H_13_O_6_ 313.0712).

### 3.6. 3-Propyl-1,6,8-trihydroxy-9,10-anthraquinone (**3**)

An orange-yellow optically inactive powder. IR (film) ν_max_ 3420, 1660, 1280 cm^−1^; UV (PDA, MeOH) λ_max_ 512 nm; ^1^H-NMR (600 MHz, CD_3_OD) δ 7.58 (1H, H-4, brs), 7.18 (1H, H-5, d, *J* = 2.3 Hz), 7.10 (1H, H-2, brs), 6.56 (1H, H-7, d, *J* = 2.3 Hz), 2.69 (2H, H-1’, t, *J* = 7.3 Hz), 1.71 (2H, H-2’, sep, *J* = 7.3 Hz), 0.99 (3H, H-3’, t, *J* = 7.3 Hz); ^13^C-NMR (150 MHz, CD_3_OD) see Tables 1 and 2; HRESIMS *m/z* found 297.0782 for [M-H]^−^ (calcd for C_17_H_13_O_5_ 297.0802).

### 3.7. 4-[(Phenylacetyl)amino]ethanesulfonic acid (**4**)

A colourless optically inactive oil. ^1^H-NMR (600 MHz, DMSO-d_6_) δ 2.53 (H_2_-1, brt, *J* = 7.3 Hz), 3.29 (H_2_-2, dt, *J* = 5.6, 7.3 Hz), 3.37 (H_2_-4, s), 7.21 (H-8, t, *J* = 7.5 Hz), 7.24 (2H, H-6 and H-10, d, *J* = 7.5 Hz), 7.28 (2H, H-7 and H-9, t, *J* = 7.5 Hz); ^13^C-NMR (150 MHz, DMSO-d_6_) δ 35.7 (t, C-2), 42.5 (t, C-4), 50.6 (t, C-1), 126.3 (s, C-8), 128.2 (2 × d, C-6, 10), 129.0 (2 × d, C-7, 9), 136.4 (s, C-5), 169.7 (s, C-3); HRESIMS *m/z* found 242.0493 for [M-H]^−^ (calcd for C_10_H_12_NO_4_S 242.0487).

### 3.8. 4-Hydroxybutanoic acid (**5**)

A colourless optically inactive oil. ^1^H (600 MHz, CDCl_3_) δ 4.35 (2H, H-4, t, *J* = 7.2 Hz), 2.50 (2H, H-2, t, *J* = 8.1 Hz), 2.26 (2H, H-3, tt, *J* = 7.2, 8.1 Hz); ^13^C (150 MHz, CDCl_3_) δ 177.8 (s, C-1), 68.5 (t, C-4), 27.8 (t, C-2), 22.1 (t, C-3); ESIMS *m/z* 103 [M-H]^−^, 85 [M-H_2_O]^−^.

## Figures and Tables

**Table 1.** Comparison of NMR data for **1** isolated in the current study (^1^H-NMR at 600 MHz, ^13^C-NMR at 150 MHz, basic frequency) with that reported previously for **1** [8–10] and with **3**.

	**^13^C δ (m)**			**^1^H δ (m,*J*Hz)**					**HMBC (6 Hz)**	**gHMBC (6 Hz)**	**1,1-ADEQUATE**

No.	**1**^a^,^10^	**1**^a^	**3**^a^	**1**^a^,^10^	**1**^a^	**1**^b^,^9^	**1**^b^	**1**^c^,^8^	**1**^a^,^10^	**1**^a^	**1**^a^
1	163.7 (s)	163.6 (s)	163.6 (s)								
2	122.4 (d)	122.3 (d)	124.7 (d)	7.20 (s)	7.24 (d, 1.4)	7.17 (brs)	7.24 (d, 1.4)	6.73 (d, 1.2)	C-4, 1’	C-1’, 1, 3, 4, 9a	C-1, 3
3	156.1 (s)	156.6 (s)	154.2 (s)								
4^d^	116.6 (d)	118.4 (d)	121.2 (d)	7.70 (s)	7.72 (d, 1.4)	7.56 (brs)	7.66 (d, 1.4)	7.16 (d, 1.2)	C-2, 10, 1’	C-1’, 1, 2, 9, 9a, 10	C-3, 4a
4a	135.3 (s)	134.9 (s)	134.7 (s)								
5^d^	113.6 (d)	110.4 (d)	109.1 (d)	7.09 (s)	7.16 (d, 2.4)	6.96 (d, 2.0)	7.02 (d, 2.5)	6.61 (d 2.0)	C-10	C-6, 7, 8, 8a, 9, 10	C-6, 10a
6^d^	160.2 (s)	166.7 (s)	166.6 (s)								
7	109.7 (d)	109.1 (d)	110.1 (d)	6.35 (d, 3.4)	6.52 (d, 2.4)	6.41 (d 2.0)	6.44 (d, 2.5)	6.02 (d, 2.0)	C-5	C-5, 6, 8, 8a	C-6
8	167.5 (s)	167.9 (s)	167.2 (s)								
8a^d^	113.9 (s)	110.5 (s)	110.5 (s)								
9	192.0 (s)	191.7 (s)	191.9 (s)								
9a^d^	118.4 (s)	115.9 (s)	115.0 (s)								
10	184.3 (s)	183.2 (s)	183.2 (s)								
10a	136.9 (s)	136.9 (s)	136.9 (s)								
1′	75.5 (d)	75.4 (d)	39.2 (t)	4.60 (m)	4.62 (t, 6.4)	4.56 (m)	4.57 (t, 6.5)	4.19 (t, 5.8)	C-2, 4	C-2’, 3’, 2, 3, 4	C-2’, 3
2′	32.6 (t)	32.6 (t)	24.8 (t)	1.73 (m)	1.75 (m)	1.65 (m)	1.63 (m)		C-3, 3′	C-1’, 3’, 3	C-1’, 3’
3′	10.3 (q)	10.2 (q)	14.0 (q)	0.94 (t, 12.5)	0.96 (t, 7.6)	0.91 (t, 8.0)	0.86 (t, 7.3)		C-1’, 2′	C-1’, 2’	C-2’

a Measured in CD_3_OD, referenced to internal solvent signals (δ 3.31 and 49.0 ppm).

b Measured in DMSO d_6_, referenced to internal solvent signals (δ2.50).

c Measured in (CD_3_)_2_CO, referenced to TMS (δ0).

d NMR resonances and coupling constants associated with atoms at these positions are not consistent with the current molecule and the previously reported rhodoptilometrin [10] being identical.

8–10 Data comes from references [8–10], see main text.

**Table 2.** ^13^C-NMR (600 MHz, CD_3_OD, 298 K) data for **1** (rhodoptilometrin) at various concentrations.

**^13^C δ (m) for 1 in CD_3_OD**						
**No.**	**Unknown^a^**	**Unknown**	**conc mg/600 μL**		**8**	**4**
			**32**	**16**		
1	163.7 (s)	163.6 (s)	163.4 (s)	163.5 (s)	163.6 (s)	163.7 (s)
2	122.4 (d)	122.3 (d)	122.2 (d)	122.2 (d)	122.3 (d)	122.3 (d)
3	156.1 (s)	156.6 (s)	156.5 (s)	156.6 (s)	156.6 (s)	156.7 (s)
**4^b^**	***116.6 (d)***	***118.4 (d)***	**118.4 (d)**	**118.4 (d)**	**118.4 (d)**	**118.4 (d)**
4a	135.3 (s)	134.9 (s)	134.5 (s)	134.7 (s)	134.8 (s)	134.8 (s)
**5^b^**	***113.6 (d)***	***110.4 (d)***	**110.4 (d)**	**110.4 (d)**	**110.4 (d)**	**110.4 (d)**
**6^b^**	***160.2 (s)***	***166.7 (s)***	**166.4 (s)**	**166.5 (s)**	**166.6 (s)**	**166.7 (s)**
7	109.7 (d)	109.1 (d)	109.0 (d)	109.0 (d)	109.0 (d)	109.1 (d)
8	167.5 (s)	167.9 (s)	167.1 (s)	167.2 (s)	167.3 (s)	167.4 (s)
**8a^b^**	***113.9 (s)***	***110.5 (s)***	**110.3 (s)**	**110.4 (s)**	**110.5 (s)**	**110.6 (s)**
9	192.0 (s)	191.7 (s)	191.5 (s)	191.7 (s)	191.8 (s)	191.9 (s)
**9a^b^**	***118.4 (s)***	***115.9 (s)***	**115.6 (s)**	**115.7 (s)**	**115.8 (s)**	**115.9 (s)**
10	184.3 (s)	183.2 (s)	182.7 (s)	182.9 (s)	183.0 (s)	183.1 (s)
10a	136.9 (s)	136.9 (s)	136.6 (s)	136.7 (s)	136.9 (s)	137.0 (s)
1′	75.5 (d)	75.4 (d)	75.4 (d)	75.4 (d)	75.4 (d)	75.4 (d)
2′	32.6 (t)	32.6 (t)	32.6 (t)	32.6 (t)	32.6 (t)	32.6 (t)
3′	10.3 (q)	10.2 (q)	10.2 (q)	10.2 (q)	10.2 (q)	10.2 (q)

a Data from reference [10].

b Highlighted rows show ppm values with largest discrepancies from values published in reference [10].

**Table 3.** GI_50_ (μM) data for **1**, and **3–5** against a series of human tumour cell lines.

**No.**	**SF-268^a^**	**MCF-7^b^**	**H460^c^**
**1**	41	21	25
**3**	72	20	25
**4**	>250	195	>250
**5**	68	32	50

a SF-268 Central nervous system-glioblastoma cells.

b MCF-7 Breast-pleural effusion adenocarcinoma cells.

c H460 Lung-large cell carcinoma cells.
